# Supplementary material for: Dual Inhibition of H3K9me2 and H3K27me3 Promotes Tumor Cell Senescence without Triggering the Secretion of SASP
Source: Int J Mol Sci. 2022 Apr 1;23(7):3911. doi: 10.3390/ijms23073911 (PMC8999616; doi:10.3390/ijms23073911)

Figure 1C

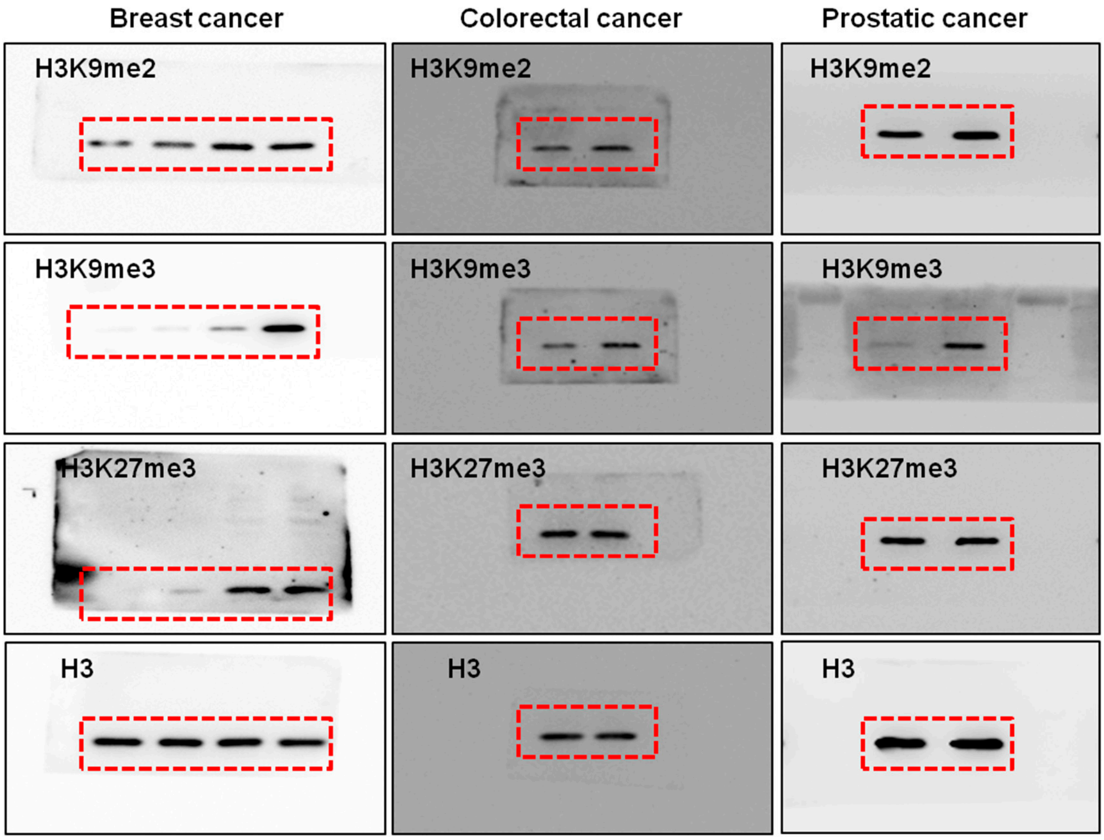

Figure 2B

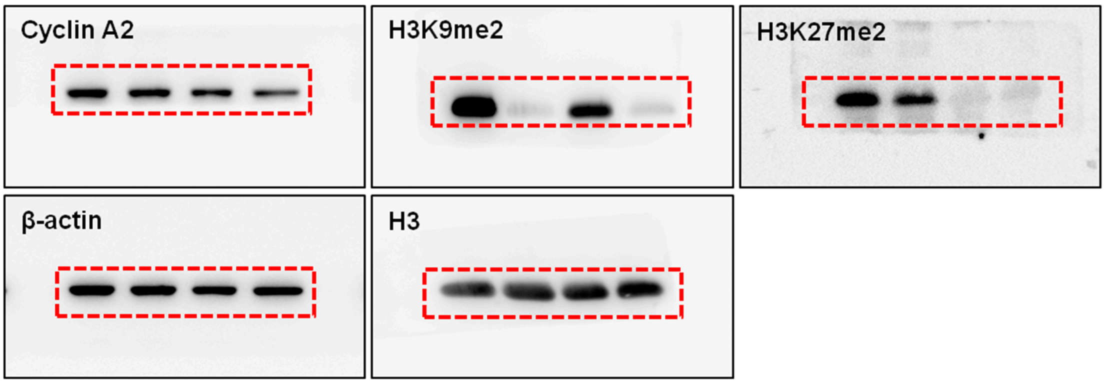

Figure 3A

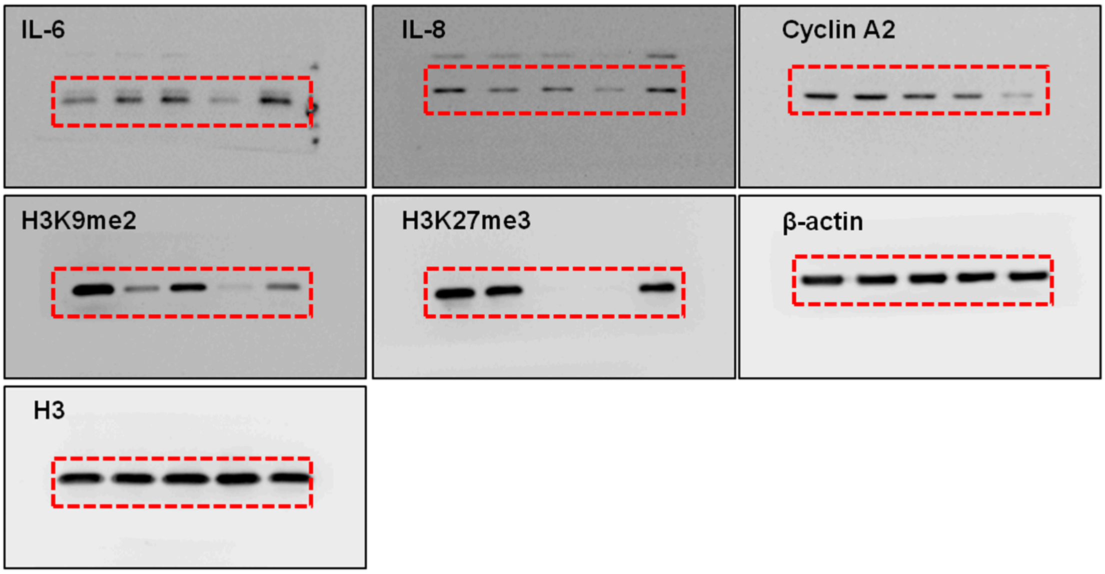

Figure 3B

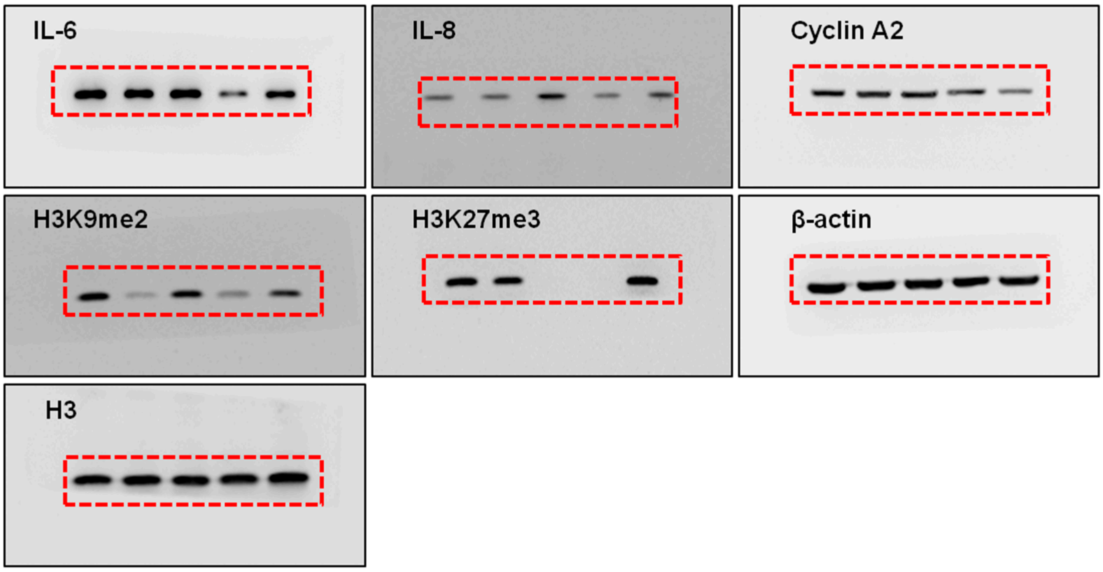

Figure 3C

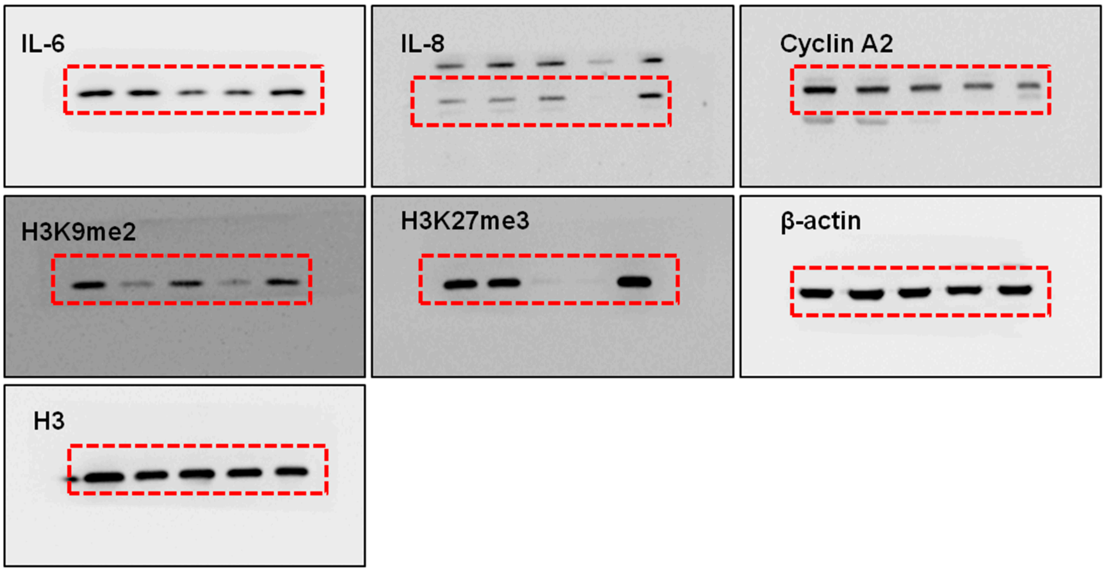

Figure 4C

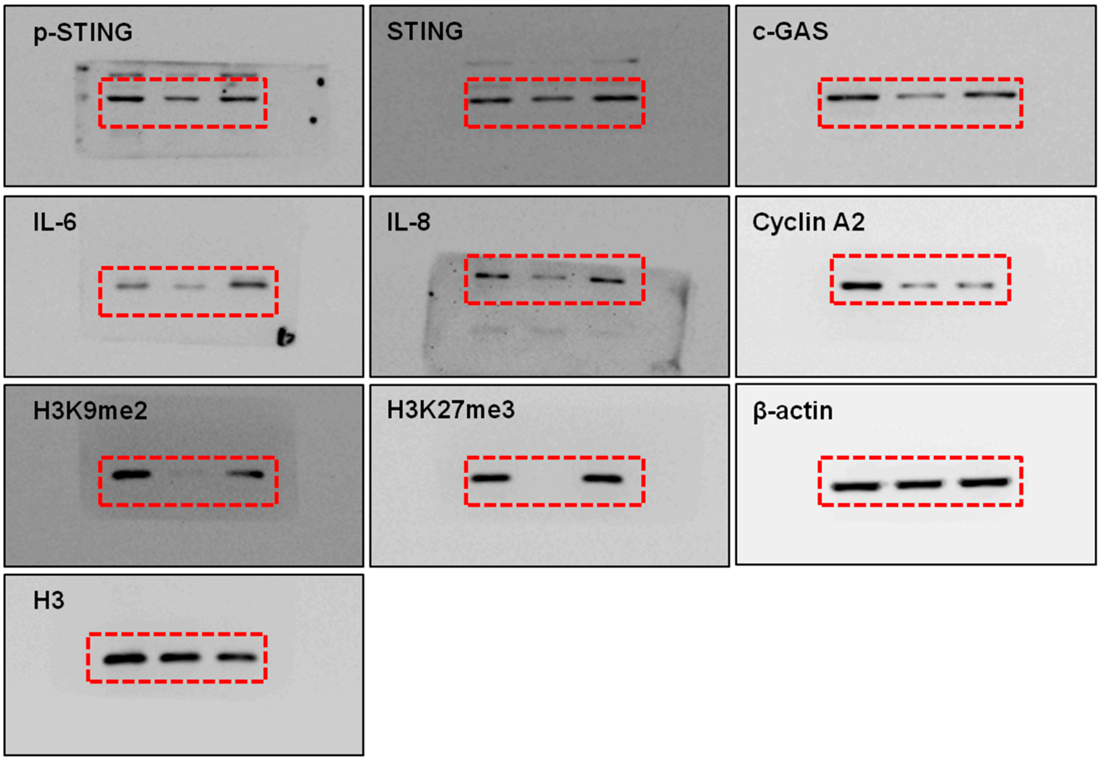

Figure 4D

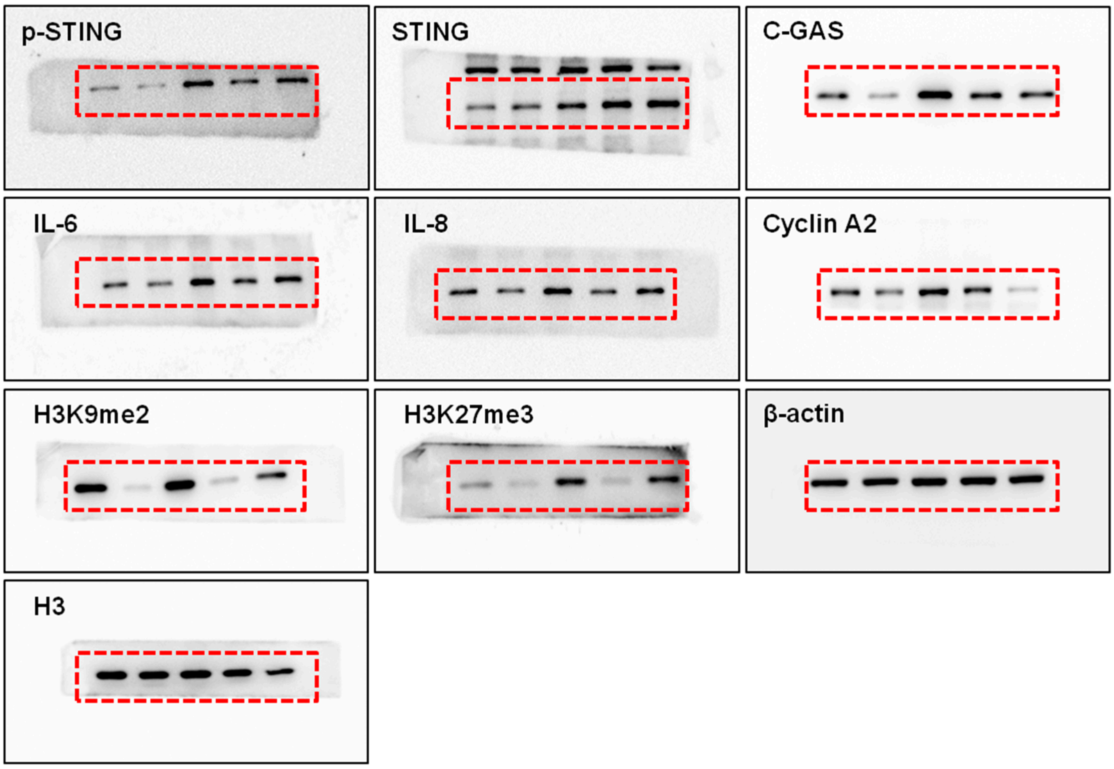

Figure S2A

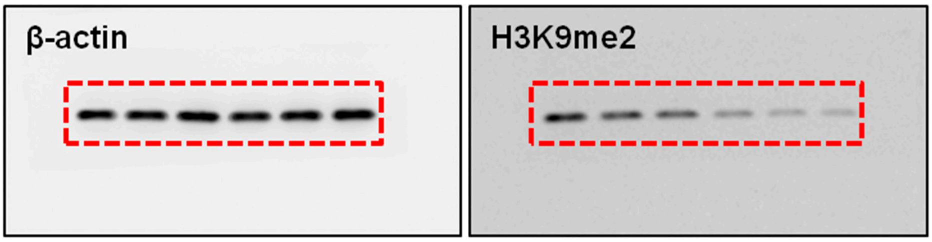

Figure S2B

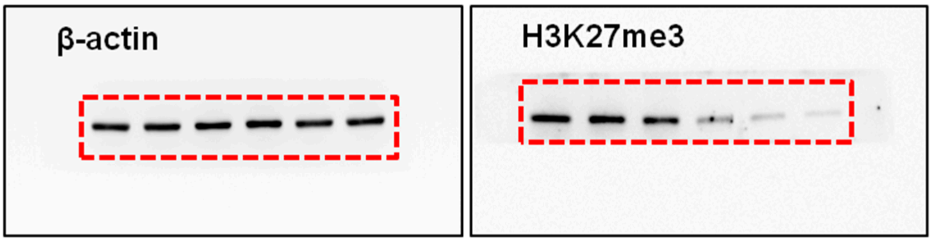

Figure S2C

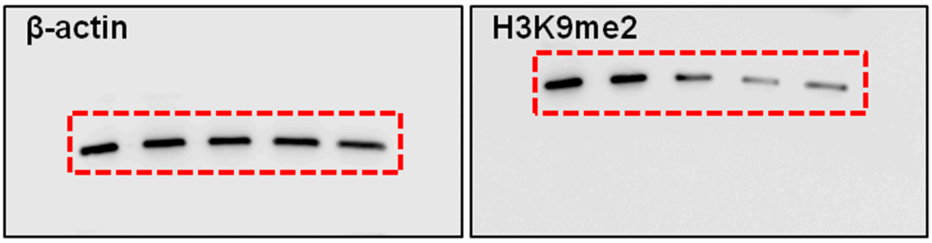

Figure S2D

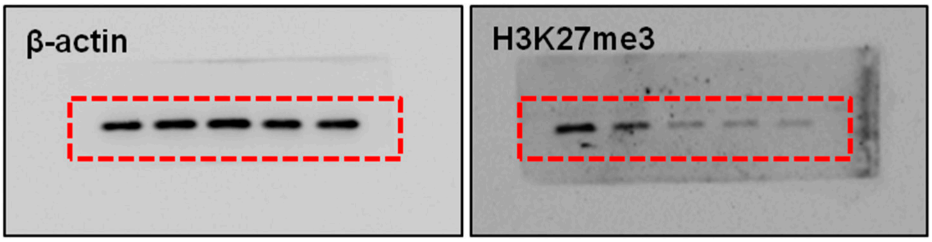

Figure S4C

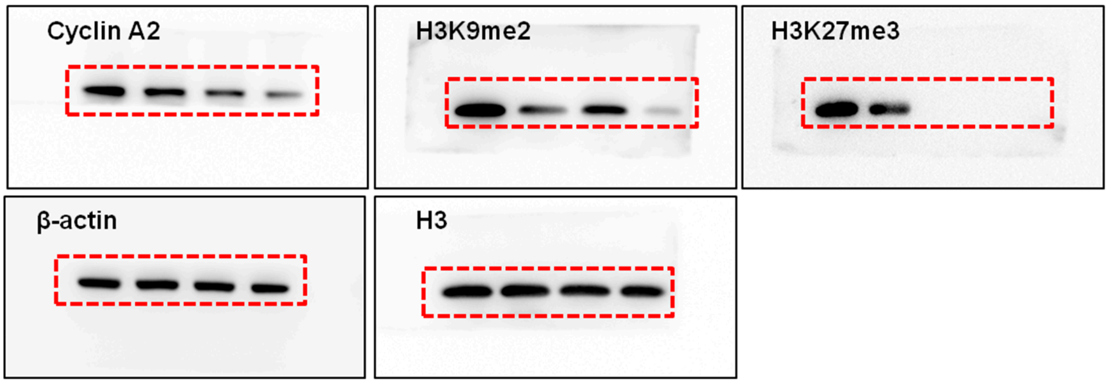

Figure S4E

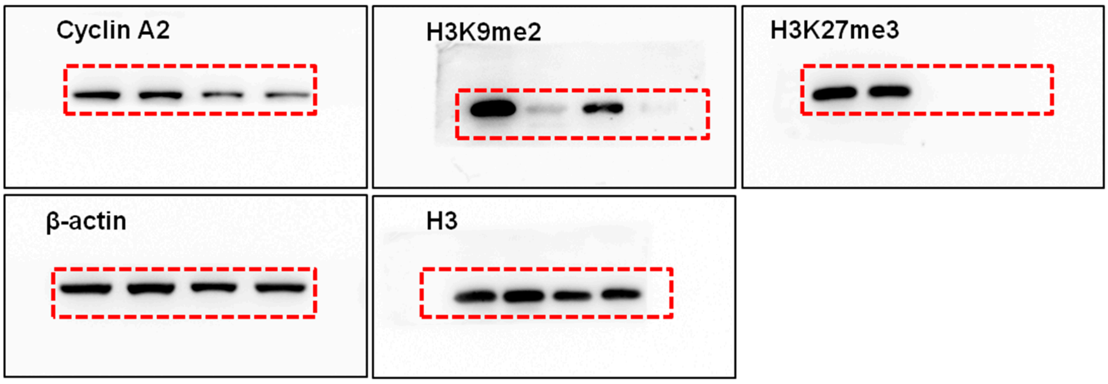

Figure S5A

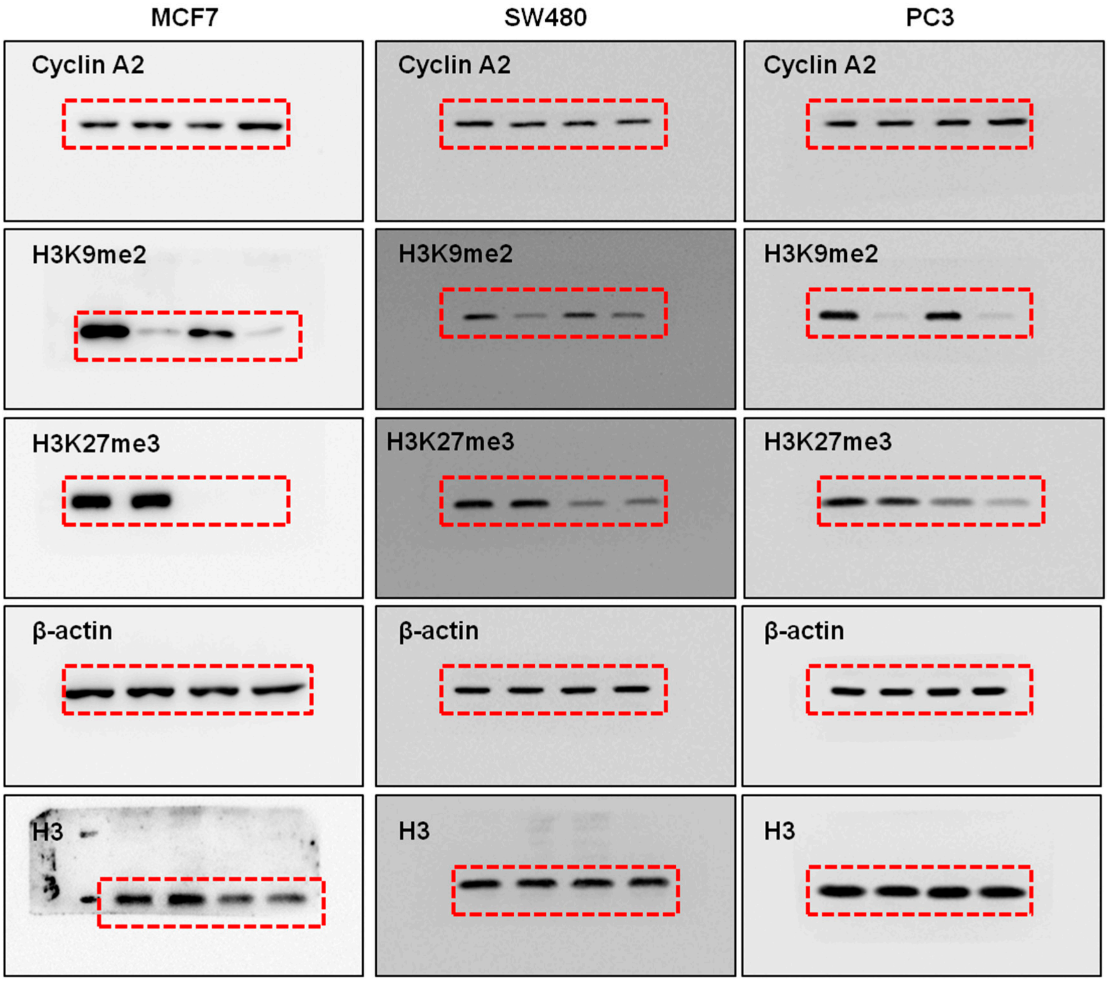

Figure S5B

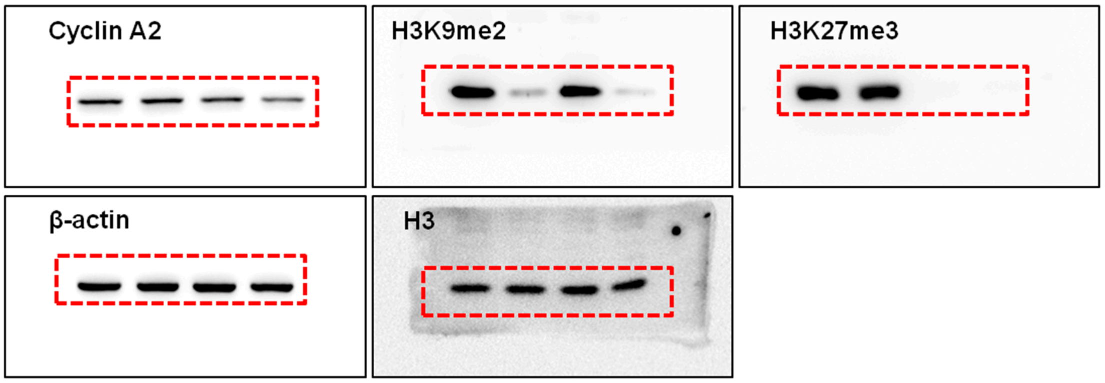

Supplement: Supplementary file 1 [file ijms-23-03911-s001.zip › original images of western blot.pdf]
