# Supplementary material for: Dual Inhibition of H3K9me2 and H3K27me3 Promotes Tumor Cell Senescence without Triggering the Secretion of SASP
Source: Int J Mol Sci. 2022 Apr 1;23(7):3911. doi: 10.3390/ijms23073911 (PMC8999616; doi:10.3390/ijms23073911)
Supplement: Supplementary file 1 [file ijms-23-03911-s001.zip › Supplementary Materials.pdf]

Supplementary Materials

Dual inhibition of H3K9me2 and H3K27me3 promotes tumor cell senescence without triggering the secretion of SASP

Zhang et al

Supplementary Figures

Figure S1: Expression of EZH2 and G9a in primary tumor (breast cancer, colon cancer and prostate cancer) samples compared with the normal samples.

Figure S2: Determination of the optimum concentration of inhibitors.

Figure S3: A combination of UNC0642 and UNC1999 blocks migration of tumor cells significantly.

Figure S4: A combination of UNC0642 and UNC1999 induces HCT116 and DU145 cellular senescence.

Figure S5: Drug sensitivity is related to tumor malignancy.

Supplementary Figure Legends

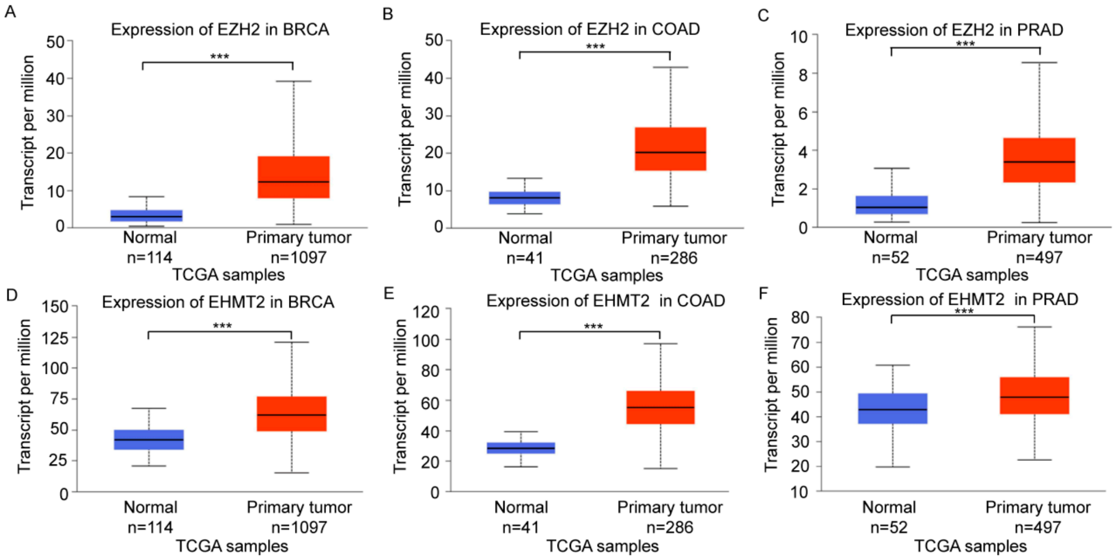

Figure S1. Expression of EZH2 and G9a in primary tumor (breast cancer, colon cancer and prostate cancer) samples compared with the normal samples, BRCA, breast invasive carcinoma; COAD, colon adenocarcinoma; PRAD, prostate adenocarcinoma. (**A-C**) Expression analysis of EZH2 in different tumor samples according to the TCGA database. (**D-E**) Expression analysis of EHMT2 in different tumor samples according to the TCGA database. $^{***}p < 0.001$.

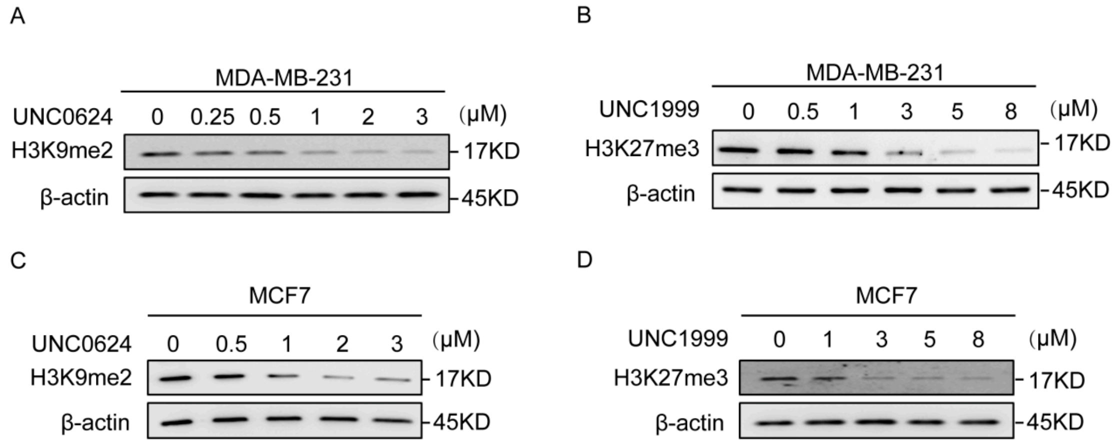

Figure S2. Determination of the optimum concentration of inhibitors. (**A**) Western blot analysis of expression of H3K9me2 in MDA-MB-231 cells treated with UNC0642 (0 μM, 0.25 μM, 0.5 μM, 1 μM, 2 μM, 3 μM) for 3 days. (**B**) Western blot analysis of expression of H3K27me3 in MDA-MB-231 cells treated with UNC1999 (0 μM, 0.5 μM, 1 μM, 3 μM, 5 μM, 8 μM) for 3 days. (**C**) Western blot analysis of expression of H3K9me2 in MCF7 cells treated with UNC0642 (0 μM, 0.5 μM, 1 μM, 2 μM, 3 μM) for 3 days. (**D**) Western blot analysis of expression of H3K27me3 in MCF7 cells treated with UNC1999 (0 μM, 1 μM, 3 μM, 5 μM, 8 μM) for 3 days.

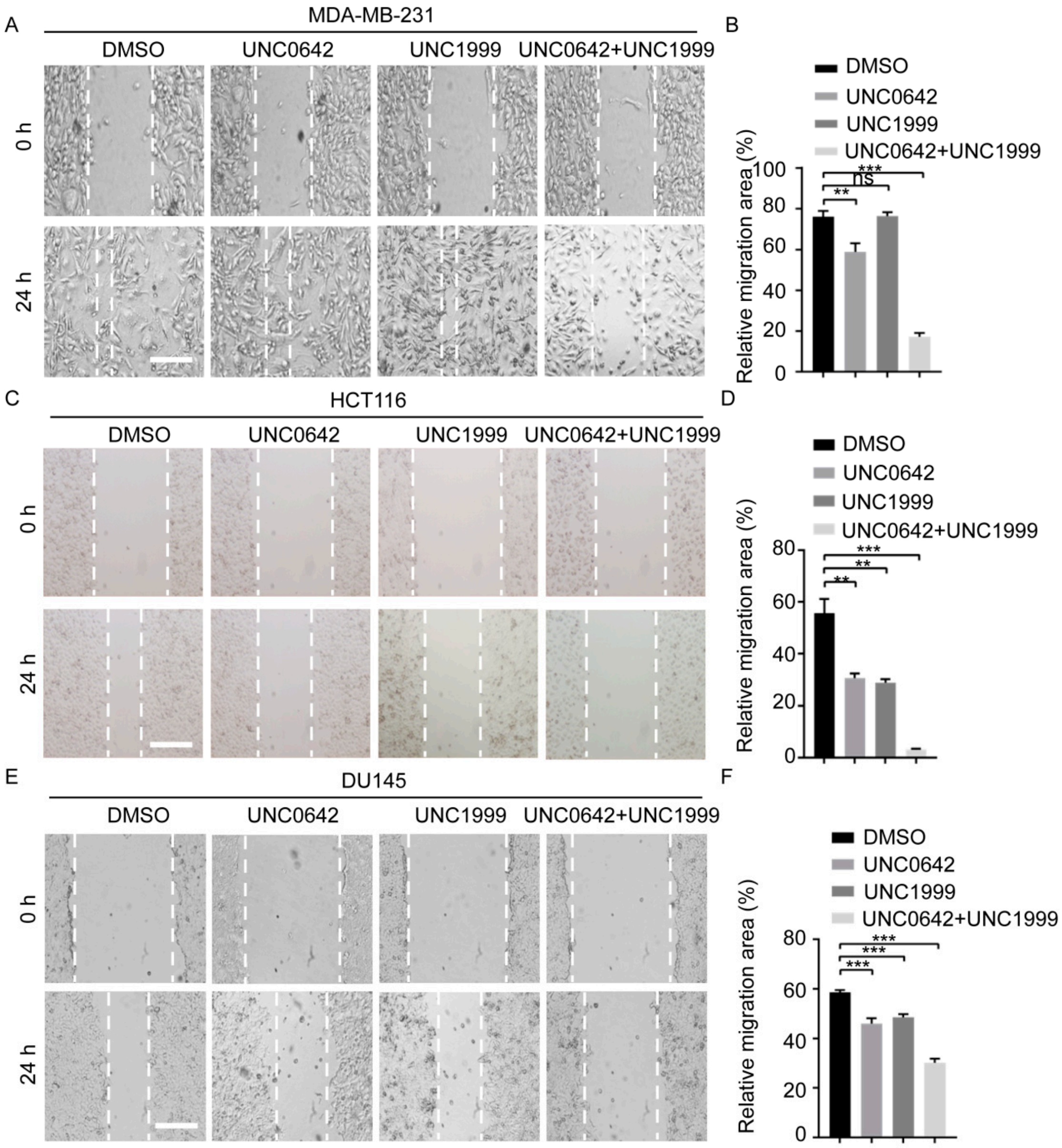

Figure S3. A combination of UNC0642 and UNC1999 blocks migration of tumor cells significantly. (**A-B**) Wound-healing assay in MDA-MB-231 and DU145 cells treated with indicated treatments. Representative micrographs of the wound-healing assay are shown (**A**) and data represent the relative migration area of cells per field, Scale bar: 100 μm (**B**). (**C-D**) Wound-healing assay in HCT116 cells treated with indicated treatments. Representative micrographs of the wound-healing assay are shown (**C**) and data represent the relative migration area of cells per field, Scale bar: 100 nm (**D**). (**E-F**) Wound-healing assay in DU145 cells treated with indicated treatments. Representative micrographs of the wound-healing assay are shown (**E**) and data represent the relative migration area of cells per field, Scale bar: 100 μm (**F**). Each experiment was repeated at least three times. Error bars, mean ± SD, ns: no significant, $^{*}p < 0.05$, $^{**}p < 0.01$, $^{***}p < 0.001$.

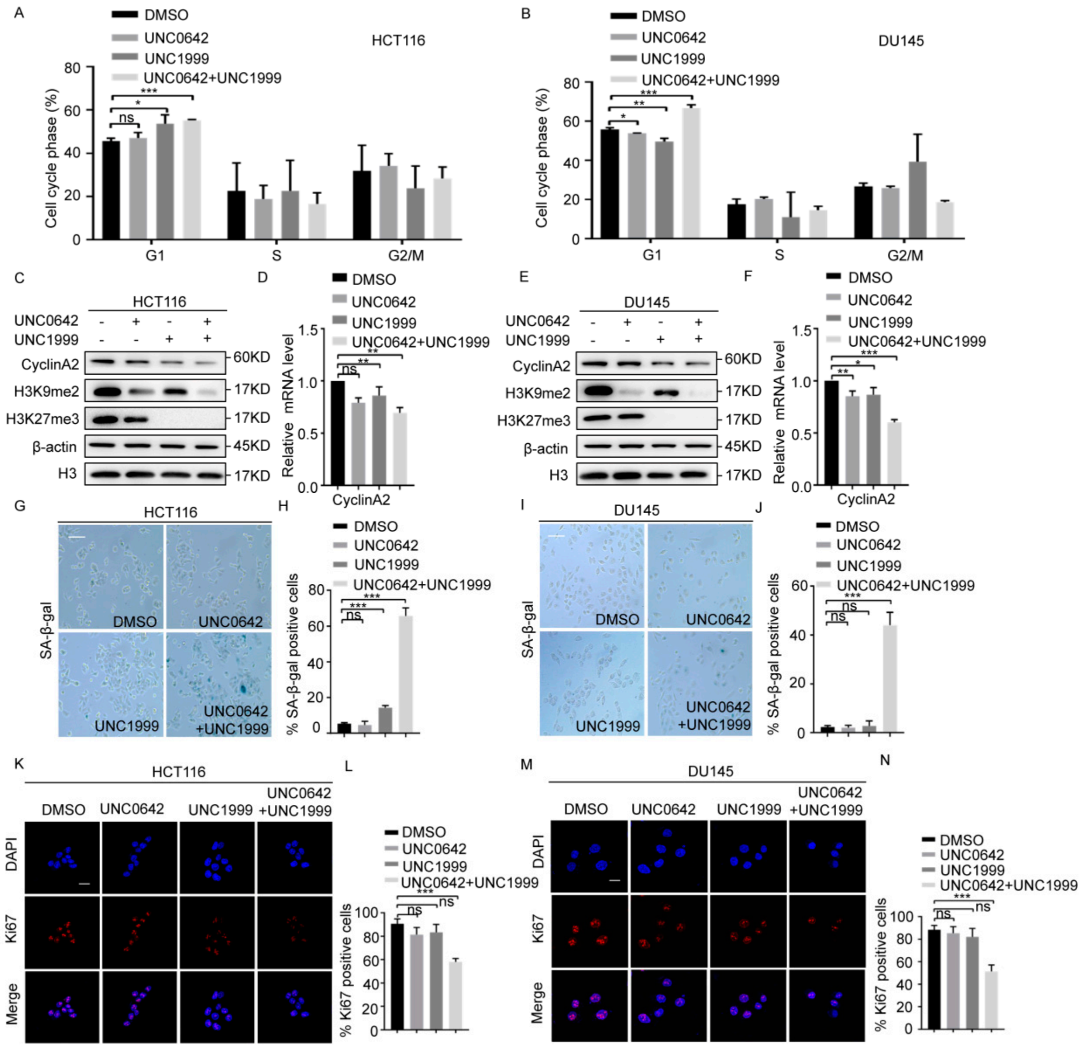

Figure S4. A combination of UNC0642 and UNC1999 induces HCT116 and DU145 cellular senescence. (**A-B**) Cell cycle analysis in HCT116 (**A**) and DU145 (**B**) cells treated with indicated treatments. (**C**) Western blot analysis of expression of CyclinA2, H3K9me2 and H3K27me3 in HCT116 cells treated with indicated treatments. (**D**) qRT-PCR analysis of the expression of CyclinA2 in HCT116 cells treated with indicated treatments. (**E**) Western blot analysis of expression of CyclinA2, H3K9me2 and H3K27me3 in DU145 cells treated with indicated treatments. (**F**) qRT-PCR analysis of the expression of CyclinA2 in HCT116 cells treated with indicated treatments. (**G-J**) SA-β-Gal staining in HCT116 and DU145 cells treated with indicated treatments. Representative images of SA-β-Gal positive cells were showed (**G** and **I**) and the percentage of the SA-β-Gal positive cells was calculated, Scale bar: 100 μm (**H** and **J**). (**K-N**) Immunofluorescence analysis of Ki67 in HCT116 and DU145 cells with the indicated treatments. Representative images of Ki67 positive cells were showed (**K** and **M**) and the percentage of the Ki67 positive cells was calculated, Scale bar: 20 μm (**L** and **N**). Each experiment was repeated at least three times. Error bars, mean ± SD, ns: no significant, $^{*}p < 0.05$, $^{**}p < 0.01$, $^{***}p < 0.001$.

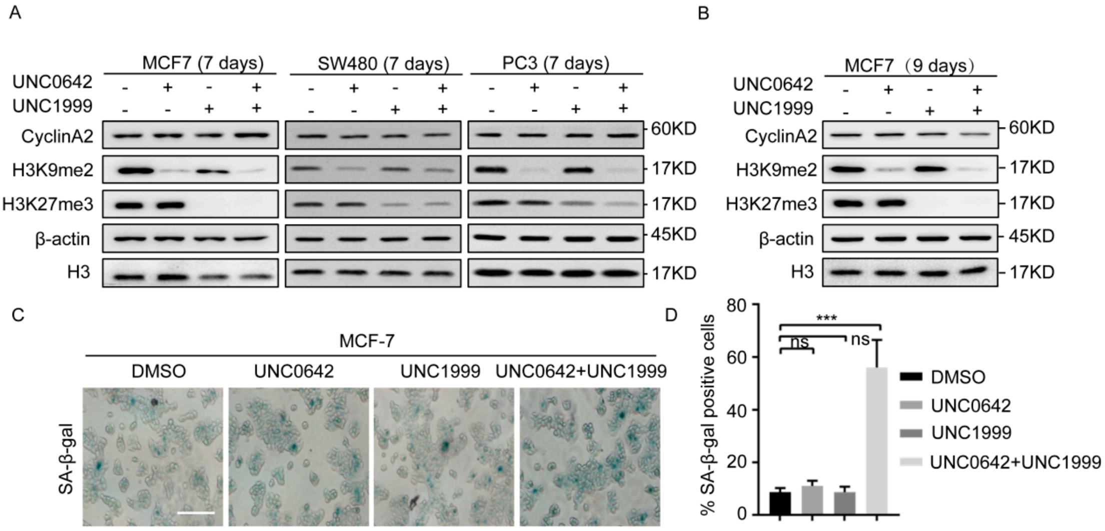

Figure S5. Drug sensitivity is related to tumor malignancy. (**A**) Western blot analysis of expression of CyclinA2, H3K9me2 and H3K27me3 in MCF7 cells, SW480 cells and PC3 cells treated with indicated treatments for 7 days. (**B**) Western blot analysis of expression of CyclinA2, H3K9me2 and H3K27me3 in MCF7 cells with indicated treatments for 9 days. (**C-D**) SA-β-Gal staining in MCF7 cells treated with indicated treatments for 9 days. Representative images of SA-β-Gal positive cells were showed (**C**) and the percentage of the SA-β-Gal positive cells was calculated, Scale bar: 100 nm (**D**). Each experiment was repeated at least three times. Error bars, mean ± SD, ns: no significant, $^{***}p < 0.001$.
